# Cleaning and Polishing Teeth

**Published:** 1872-11

**Authors:** L. A. Augspath


### Cleaning and Polishing Teeth.

BY L. A. AUGSPATH.

*Read before the Tennesee State Dental Society.*

There is doubtless in the whole range of causes nothing which exerts a greater influence upon the profession of dentistry than the neglect on the part of the mass of mankind, of proper care of the teeth and mouth. Want of which care is beyond all question the source of many of the diseases and evils to which the teeth are subject. There is probably nothing which more claims the attention of the intelligent dentist than the subject of deposits, including the calcareous formation, usually denominated *tartar*, *green or brown stain*, and all those impurities on the teeth which are produced by *neglect*, tobacco, and other similar causes.

It is well understood that there are different varieties of tartar, determined by color, composition and consistency; but all produced by the same cause, and resulting as a precipitate of the saliva in connection possibly with the deposits of the mucus.

Persons of all ages are subject to the deposits of tartar, although it seldom appears before children have produced their-*six* year molars, but continues to be formed until the most advanced age, when the teeth may be nearly covered with it.

Some deposit tartar through life, while others are exempt until some great constitutional change takes place, when it is rapidly eliminated.

Green and brown stain doubtless are caused almost exclusively by the mucus—tartar by the saliva. The stain is not a formation on the tooth, but enters into its composition and tends to produce decay. Tartar is an exterior formation on the surface of the tooth, and serves rather to prevent decay.

In the treatment of the teeth, as with all other diseases, physical or moral, there is much truth in the old maxim—"an ounce of prevention is better than a pound of cure." The most potent of all preventions of tooth disease is cleanliness of the teeth and mouth. The mouth should be always thoroughly cleansed with luke-warm water upon first rising in the morning, immediatey after each meal, and just before retiring at night. The custom which prevails in some portions of France and England, of thoroughly rinsing the mouth with aromatic water, is highly beneficial and is well worthy of more general adoption. Its tendency is not only to preserve the teeth, but also to give the voice a clearness and pureness of tone seldom otherwise acquired.

By the custom of thoroughly cleansing the mouth with brush, tepid water, soap and toothpick, the formation of tartar may be entirely prevented.

Every mother should consider it her duty to see that the mouths and teeth of her offspring are thoroughly cleansed. This habit becoming fixed in the child, their comfort will ever after demand perfect cleanliness.

After an experience as a practitioner of over fifteen years, I would suggest that too much stress cannot be laid upon perfect cleanliness as a preventive of diseases of the teeth and mouth.

There are two methods of removing salivary calculus from the teeth. The one chemically, by decomposing the deposit by the use of an acid; the other mechanically, by scaling and scraping with appropriate instruments. The former should never be resorted to, as the chemical action of the acid does not stop with the decomposition of the calcareous deposit, but by the same affinity attack the tooth itself, and with almost equal readiness destroy it.

The removal of tartar by the second method does not involve a very great amount of skill, but with suitable instruments is easily performed. To accomplish the operation with perfect success, appliances and instruments of various forms and curves are necessary, adapted and adjusted to the various shapes snd situations to be operated on. The process should in every instance be performed with the most perfect thorougness.

All instruments should be very sharp, but with the cutting edge removed. The blade of the instrument should be applied at a slightly obtuse angel with the surface of the tooth beyond the edge of the tartar next to the gum, and, passing under the deposit, thus scale it off to the point, in such a manner as not to roughen, scratch or abrade the enamel. Tartar which is deposited on proximal surfaces of the teeth is to be carefully noticed and removed with instruments with very thin blades.

When the thick deposits have been removed the surface should then be carefully and gently scraped, so as thoroughly to clean off every particle of the tartar, and afterward fully and completely polished with fine pumice, Arkansas or rotten stone, and finished by carefully burnishing,

To effect a complete remedy for green or brown stain it is frequently imperative to combine therapeutic treatment with operative—yet the latter must be perfect and thorough.

There are various methods of accomplishing the mechanical operation. Where the erosoin is but slight, Arkansas or pumice stone will often be found sufficient, but should this fail, recourse must be had to the file, and afterward using the pulverized stone. But, gentlemen, you doubtless understand the practical treatment as well as I.

It is my purpose to direct attention more particularly to the necessity of thoroughness in cleansing the teeth. Doubtless it is needless for me to call your attention to the direct agency of bodily ailments in producing and increasing various diseases of the teeth, and the reactive influence which dead and decayed teeth, as well as deposits and impurities of the teeth and mouth, exert upon the general health.

Some of our professional brethren appear to regard the operation of cleaning the teeth as unimportant, and look upon the consequences of its neglect with great carelessness.

Others seem to be disinclined to give the required time to the discharge of this duty. From these causes the operation is not generally more than half performed—a little scratching and scraping certainly is given, but in many instances the mouth and teeth are left in a worst condition than before they were touched, and "little being given," little is generally "required" for their so-called surfaces. Frequently we hear of operators whose prices for cleaning teeth range from fifty cents to one dollar; while I am convinced, gentlemen, that you will second my assertion, that often a fee of from ten to fifty dollars is not a disproportionate recompense for the thorough and faithful performance of this important work. Sometimes days, weeks, and even months of constant treatment are necessary to thoroughly clean and bring into a proper healthy condition some bad cases; and it is very seldom that a mouth can be properly treated and thoroughly cleaned at one sitting.

Most cases which require careful cleaning are very disagreeable, and many are positively disgusting. The popular mind seems to be lamentably ignorant on the subject of the proper care of the mouth; and generally if the practioner does not positively insist upon thorough cleanliness of the teeth, the patent will very complacently neglect the matter, both on account of the expense and the unpleasantness of the operation. But, gentlemen, however disagreeable this duty may be, it is really obligatory and binding upon us to devote to the matter all the time and labor which may be necessary to effect a complete and radical cure.

We should ever impress upon our patients the necessity of careful and frequent attention to this subject. And when we undertake to clean teeth we should never be satisfied with half performing a work, but it should be completely and thoroughly effected. Our duty to our profession and to our own personal reputation demands that in the case of our regular patients we should not permit the subject of cleanliness of the teeth and mouth to be neglected, even though we are obliged to treat them gratuitously. Local treatment will many times prove insufficient even in cases which are not complicated with constitutional diseases, as syphilis, scrofula, and the like, therefore every dentist should qualify himseif to bestow this treatment in his own person rather than refer his patient to the practitioner of medicine.

This is an important duty if we would uphold and support the true dignity of our profession, and demonstrate to the world the validity of our claim to be considered an alleviating and healing profession.

Gentlemen, if we take into consideration the deplorable consequences of the neglect or half treatment of this disease, can we but wonder at the indifference with which some members of the profession regard its progress?

We find as its results, a long list of painful and appalling diseases; and it is not unfrequently the case that its influence destroys the health, and undermines the constitution to such an extent that an untimely grave can be traced to this neglect.

Then let us in our practice be very careful to be unmistakably thorough in our work as practitioners, and neglect no means of impressing upon the minds of our patients their dnty in this regard.

